# The Efficacy and Safety of Celecoxib for Pain Management After Total Knee Arthroplasty: A Systematic Review and Meta-Analysis of Randomized Controlled Trials

**DOI:** 10.3389/fsurg.2022.791513

**Published:** 2022-01-28

**Authors:** Xiaoyuan Geng, Shangyou Zhou, Xiaoyan Zhang, Xi Liu, Xu Cheng, Lihua Jiang, Donghang Zhang

**Affiliations:** ^1^Department of Anesthesiology, The Third Affiliated Hospital of Zhengzhou University, Zhengzhou, China; ^2^Department of Anesthesiology, West China Hospital of Sichuan University, Chengdu, China

**Keywords:** total knee arthroplasty, celecoxib, meta-analysis, pain management, randomized controlled trials

## Abstract

**Background:**

This study aimed to determine the efficacy and safety of celecoxib for pain management after total knee arthroplasty (TKA).

**Methods:**

PubMed, EMBASE, Web of Science, and the Cochrane Central Register of Controlled Trials (CENTRAL) were searched to identify eligible randomized controlled trials (RCTs) that compared celecoxib with a placebo in term of pain control efficacy after TKA. Primary outcomes included pain scores at 24, 48, and 72 h after TKA. Secondary outcomes included the active range of motion (ROM) at 24, 48,72 h, and 7 days postoperatively, morphine consumption over 72 h after TKA, incidence of postoperative nausea and vomiting (PONV), and total blood loss after surgery. Data analysis was conducted using RevMan version 5.3.

**Results:**

Five RCTs involving 593 participants were included in the study. Compared with a placebo, celecoxib significantly reduced visual analog scale (VAS) scores at rest at 24 h [mean difference (MD) = −0.72; 95% confidence interval (CI), −1.27 to −0.17; *I*^2^ = 82%; *P* = 0.01], 48 h (MD = −1.51; 95% CI, −2.07 to −0.95; *I*^2^ = 0%; *P* < 0.00001), and 72 h (MD = −1.30; 95% CI, −2.07 to −0.54; *I*^2^ = 82%; *P* = 0.0009) after TKA, decreased morphine consumption over postoperative 72 h (MD = −0.73; 95% CI, −0.96 to −0.51; *I*^2^ = 96%; *P* < 0.00001), and increased active ROM at 48 h (MD = 13.23; 95% CI, 7.79 to 18.67; *I*^2^ = 0%; *P* < 0.00001), 72 h (MD = 6.52; 95% CI, 4.95 to 8.10; *I*^2^ = 68%; *P* < 0.00001), and 7 days (MD = 7.98; 95% CI, 3.64 to 12.31; *I*^2^ = 68%; *P* = 0.0003) after the operation. No significant difference was found in the active ROM at 24 h (MD = 7.60; 95% CI, −6.14 to 21.34; *I*^2^ = 94%; *P* = 0.28) and the incidence of PONV after surgery [risk ratio (RR) = 0.66; 95% CI, 0.40 to 1.09; *I*^2^ = 0%; *P* = 0.11].

**Conclusion:**

The administration of celecoxib is an effective and safe strategy for postoperative analgesia after TKA.

## Introduction

Total knee arthroplasty (TKA) is associated with moderate to severe postoperative pain, which contributes to the demand for analgesics and the inability to participate in postoperative rehabilitation, which leads to delayed recovery and hospital discharge (1–5). The primary goal of postoperative analgesia is to reduce both pain and the need for opioid prescriptions, thereby reducing the rate of occurrence of opioid-related adverse events. The current strategies for postoperative pain after TKA include administration of oral analgesics, periarticular injections of local anesthetics, peripheral nerve blocks, and multimodal pain regimens (6).

Celecoxib is a selective cyclooxygenase (COX)-2 inhibitor and an effective analgesic for acute postoperative pain (7). One recent study showed that early administration of celecoxib after TKA significantly reduced early visual analog scale (VAS) pain scores, improved sleep quality, and active knee flexion angles (8). However, Stepan et al. reported that celecoxib had no effect on postoperative pain and opioid consumption in patients who underwent hand surgery (9). Therefore, it is worthwhile to conduct a systematic review and meta-analysis to synthesize evidence regarding the effect of celecoxib on postoperative pain management after TKA.

## Methods

This systematic review and meta-analysis was conducted in accordance with the PRISMA guidelines (10).

### Search Strategy

Two authors (XG and DZ) independently searched PubMed, the Cochrane Central Register of Controlled Trials (CENTRAL), EMBASE, and Web of Science from the first record to April 30, 2021. The search strategy was conducted using the following keywords: celecoxib, postoperative pain, and TKA. The reference lists of relevant studies were also checked to identify other potentially eligible studies.

### Eligibility Criteria

The inclusion criteria were as follows: (a) the studies were randomized controlled trials (RCTs); (b) the participants of the studies were adult patients scheduled to undergo primary TKA due to degenerative arthritis of the knee joint; (c) the studies focused on the comparison between celecoxib and placebo for pain management of TKA; and (d) any of the following outcomes of interest were reported: perioperative VAS scores, range of motion (ROM), morphine consumption, and side effects (e.g., nausea, vomiting, and blood loss).

### Data Exaction and Outcome Measures

The following information was extracted: author, number of patients, age, sex, body mass index, duration of surgery, type of anesthesia, and outcomes. Additional data were obtained from the corresponding authors via e-mail, as necessary. Disagreements were resolved by discussion with another author. The primary outcomes were pain scores at postoperative 24, 48, and 72 h. Secondary outcomes included the ROM at postoperative 24, 48 h, and 7 days; morphine consumption over 72 h postoperatively, the incidence of postoperative nausea and vomiting (PONV), and the estimated blood loss.

Pain scores were evaluated using the VAS (VAS 0, no pain; VAS 10, the worst unbearable pain). When continuous data were presented using the median and range, we attempted to contact the corresponding author to obtain the original data. If there was no response, the median and range were converted to the mean and standard deviation (11). And two intervention groups in one study were merged into a single intervention group (12). If pain scores were not reported at rest or during movement, we tried to contact the corresponding author; if there was no response, scores were assumed to be pain scores at rest.

### Study Selection

Two authors (XG and DZ) independently reviewed the studies identified. The full text of the relevant articles was reviewed after screening their titles and abstracts. Disagreements were resolved by discussion with another author (SZ).

### Risk of Bias Assessment

Two authors (XG and DZ) independently assessed the quality of included studies using the Cochrane Collaboration's tool (12), which comprises seven items: (a) random sequence generation (selection bias), (b) allocation concealment (selection bias), (c) blinding of participants and personnel (performance bias), (d) blinding of outcome assessment (detection bias), (e) incomplete outcome data (attrition bias), (f) selective reporting (reporting bias), and (g) other bias. The estimated risk bias for each item was rated as “low risk,” “unclear risk,” or “high risk.” Disagreements were resolved by discussion with another author (SZ).

### Statistical Analysis

All statistical analyses were performed using RevMan version 5.3. Mean differences (MD) and 95% confidence intervals (CIs) were used to construct forest plots of continuous data. Risk ratios (RRs) and 95% CIs were used to construct forest plots of dichotomous data. A random-effects model was used for all analyses due to clinical heterogeneity. *I*^2^-value was used to determine the level of heterogeneity. And significant heterogeneity was considered when the *I*^2^ was > 50%. The threshold for statistical significance was set at *p* < 0.05.

## Results

### Characteristics of the Included Studies

Initially, 170 potentially relevant studies were identified, of which 91 duplicates were removed. Of the 79 records left, 67 were excluded after screening the titles and abstracts, leaving 12 potentially relevant studies for full-text review. Finally, five RCTs involving 593 patients were included in the meta-analysis (8, 13–16).

**Figure 1:**
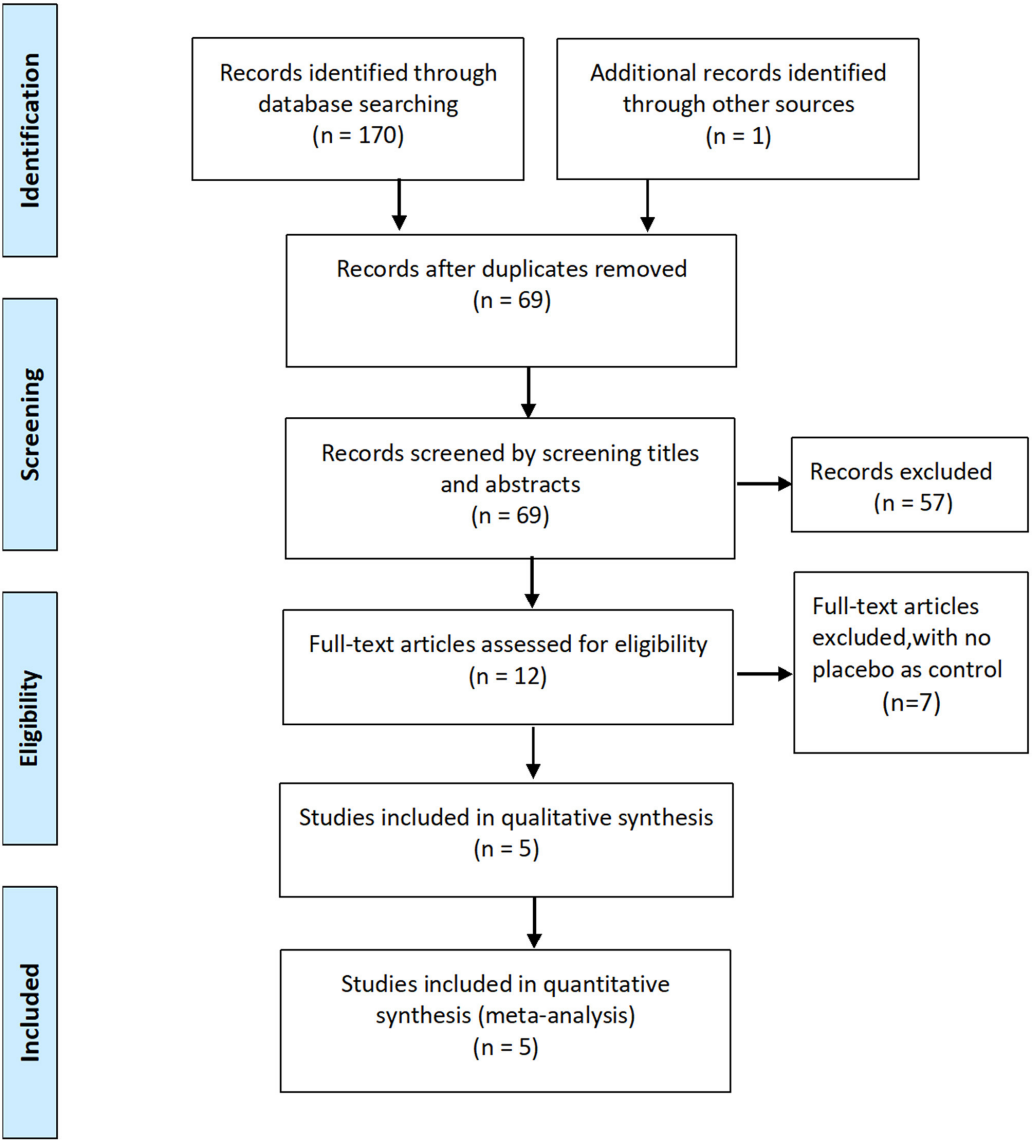
Flow diagram of study selection.

**Figure 2:**
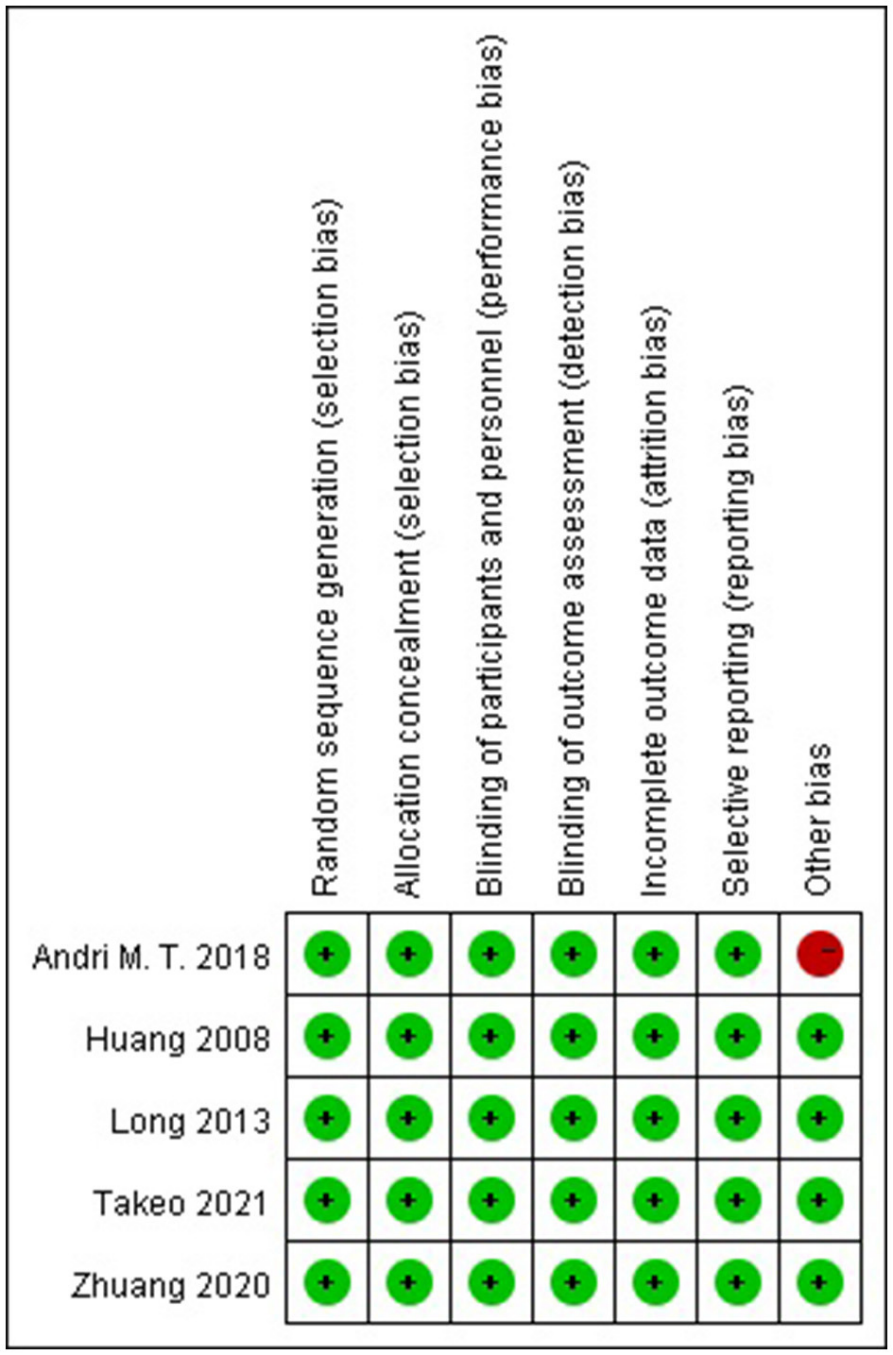
Risk of bias of included studies.

The characteristics of the included RCTs are listed in Table 1. The flow diagram of the study selection process is shown in Figure 1. The risk of bias of the included studies is shown in Figure 2.

**Table 1:** Characteristics of the included trials.

**Study**	**Group**	**Treatment**	**Anesthesia**	**Postoperative analgesia**	**Outcomes**
Gong et al., (13)	Celecoxib (*n* = 98) Control (*n* = 49)	After the surgery 150 mg, b.i.d., PO 14 days or placebo	No details provided	PCA morphine if needed	VAS pain scores at rest and ambulation, ROM, morphine consumption, blood loss, PONV, and extremities myasthenia
Lubis et al., (15)	Celecoxib (*n* = 20) Control (*n* = 10)	One hour before the operation, 400 mg or 3 days before the surgery, 200 mg, b.i.d. or placebo	Epidural anesthesia	Two hours after the surgery, paracetamol 1,000 mg, and PCA morphine if needed	Morphine consumption, VAS, knee function outcome, mobilization
Zhuang et al., (16)	Celecoxib (*n* = 123) Control (*n* = 123)	Four days after the surgery, up to 6 weeks. 200 mg celecoxib, b.i.d., or placebo	General anesthesia	PCA with morphine after the surgery and end at 24 h after the surgery, Tramadol on patient's demand or when VAS ≥3	Cumulative opioid consumption at 2 weeks post-operation, the Knee Society Score, patient-reported outcomes, the cumulative opioid consumption
Huang et al., (14)	Celecoxib (*n* = 40) Control (*n* = 40)	400 mg celecoxib, PO, 1 h before the surgery, and 200 mg every 12 h, over the first five post-operative days or placebo	Spinal anesthesia	PCA morphine	VAS, ROM, total opioid consumption, PONV
Mammoto et al., (8)	Celecoxib (*n* = 47) Control (*n* = 43)	400 mg of celecoxib 2 h after TKA, followed 6 h later by 200 mg of celecoxib or only 400 mg of celecoxib the second day after surgery	General anesthesia	PCA fentanyl	VAS of the second day after surgery, sleep quality, active ROM, VAS on postoperative days 1–7, total fentanyl consumption, PONV

*VAS, visual analog scale; ROM, range of motion; PCA, patient-controlled analgesia; PONV, postoperative nausea and vomiting*.

The included studies, which were performed in China, Japan and Indonesia, were published between 2008 and 2021. Among the five included studies, three reported VAS scores at 24 h after TKA (8, 13, 14), three reported VAS scores at 48 h after TKA (8, 13, 16), three reported VAS scores at 72 h after TKA (13, 14, 16), three reported morphine consumption over 72 h postoperatively, (13, 15, 16), two reported active ROM at 24 h after TKA (14, 16), three reported active ROM at 48 h after TKA (8, 13, 16), three (13, 14, 16) reported active ROM at postoperative 72 h, two reported active ROM at 7 days after TKA (8, 14), two (14, 16) reported the incidence of PONV, and one (14) reported total blood loss after TKA. The main outcomes of the included studies are summarized Table 2.

**Table 2:** Main outcomes of each included studies.

		**Gong et al., (13)**	**Lubis et al., (15)**	**Zhuang et al., (16)**	**Huang et al., (14)**	**Mammoto et al., (8)**
VAS scores at 24 h	Celecoxib	4.2 (1.89)	2.3 (0.28)	NR	NR	3.8 (3.6)
	Placebo	4.3 (1.98)	4 (2.22)	NR	NR	6.2 (3.1)
VAS scores at 48 h	Celecoxib	NR	1.5 (0.57)	NR	2.13 (1.68)	1.8 (2.3)
	Placebo	NR	3 (1.48)	NR	3.43 (1.5)	3.7 (2.3)
VAS scores at 72 h	Celecoxib	3.56 (1.59)	1.1(0.11)	NR	2.13 (1.68)	NR
	Placebo	4.24 (1.61)	3 (0.74)	NR	3.43 (1.5)	NR
Morphine consumption over postoperative 72 h	Celecoxib	NR	10.25 (2.18)	28.63 (14.63)	27.6 (11.9)	NR
	Placebo	NR	30.2 (5.31)	59.5 7(43.15)	24.6 (14.6)	NR
Active ROM at 24 h	Celecoxib	40.16 (2.27)	NR	NR	40.8 (17.3)	NR
	Placebo	39.2 (2.35)	NR	NR	25.8 (11.5)	NR
Active ROM at 48 h	Celecoxib	NR	45 (28.33)	NR	60.7 (18.1)	90 (17.78)
	Placebo	NR	30 (22.22)	NR	45 (17.3)	80 (22.22)
Active ROM at 72 h	Celecoxib	62.3 (7.42)	75 (23.31)	NR	77.7 (15.1)	NR
	Placebo	56.36 (2.56)	60 (7.5)	NR	64.3 (16.9)	
Active ROM at 7 days	Celecoxib	82.62 (2.45)	NR	NR	NR	106 (11.85)
	Placebo	76.26 (2.32)	NR	NR	NR	95 (12.59)
Incidence of PONV (n/N)	Celecoxib	11 (98)	NR	NR	11 (40)	NR
	Placebo	8 (49)	NR	NR	17 (40)	NR
Total blood loss after TKA	Celecoxib	746 (227)	NR	NR	NR	NR
	Placebo	780 (283)	NR	NR	NR	NR

*VAS, visual analog scale; ROM, range of motion; PCA, patient-controlled analgesia; PONV, postoperative nausea and vomiting; TKA, total knee arthroplasty; NR, not reported; SD, standard deviation. Data were present as mean and standard deviation*.

## Results of the Meta-Analyses

### VAS Scores at Rest at Postoperative 24 h

**Figure 3:**
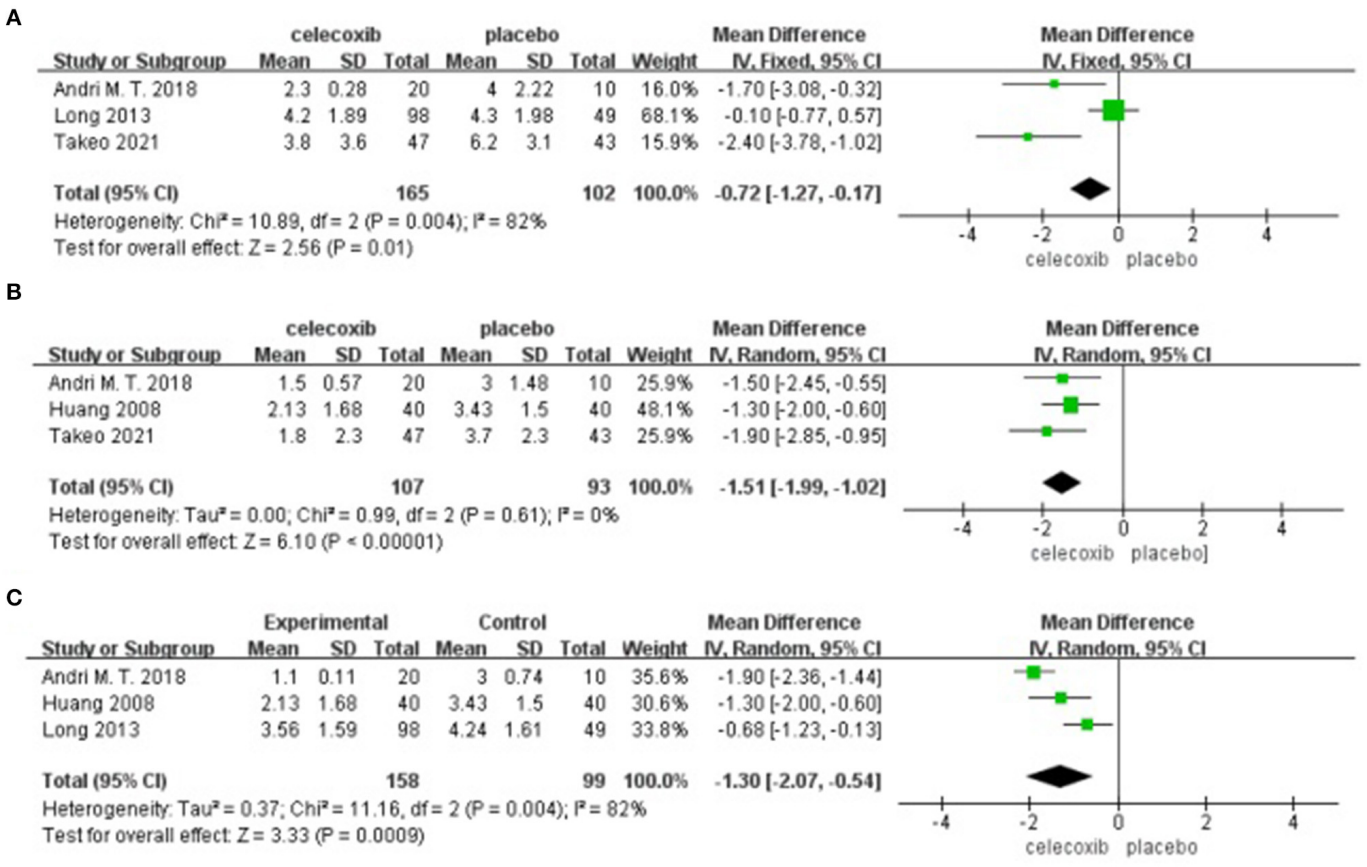
Forest plot for the meta-analysis of VAS scores at rest at postoperative **(A)** 24 h, **(B)** 48 h, and **(C)** 72 h.

Three studies (8, 13, 14) reported VAS scores at rest at postoperative 24 h. Pooled results showed a significant reduction in VAS scores at rest at 24 h in the celecoxib group compared with the placebo group (MD = −0.72; 95% CI, −1.27 to −0.17; *I*^2^ = 82%; *P* = 0.01) (Figure 3A).

### VAS Scores at Rest at Postoperative 48 h

Three studies (8, 13, 16) reported VAS scores at rest at postoperative 48 h. Pooled results showed a significant reduction in VAS scores at rest at 48 h in the celecoxib group compared with the placebo group (MD = −1.51; 95% CI, −2.07 to −0.95; *I*^2^ = 0%; *P* < 0.00001) (Figure 3B).

### VAS Scores at Rest at Postoperative 72 h

Three studies (13, 14, 16) reported VAS scores at rest at postoperative 72 h. Pooled results showed a significant reduction in VAS scores at rest at 72 h in the celecoxib group compared with the placebo group (MD = −1.30; 95% CI, −2.07 to −0.54; *I*^2^ = 82%; *P* = 0.0009) (Figure 3C).

### Morphine Consumption Over Postoperative 72 h

**Figure 4:**
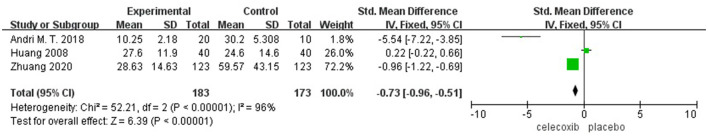
Meta-analysis of morphine consumption over postoperative 72 h.

Three studies (13, 15, 16) reported morphine consumption over 72 h postoperatively. Pooled results showed a significant reduction in morphine consumption over 72 h in the celecoxib group compared with the placebo group (MD = −0.73; 95% CI, −0.96 to −0.51; *I*^2^ = 96%; *P* < 0.00001) (Figure 4).

### Active ROM at Postoperative 24 h

**Figure 5:**
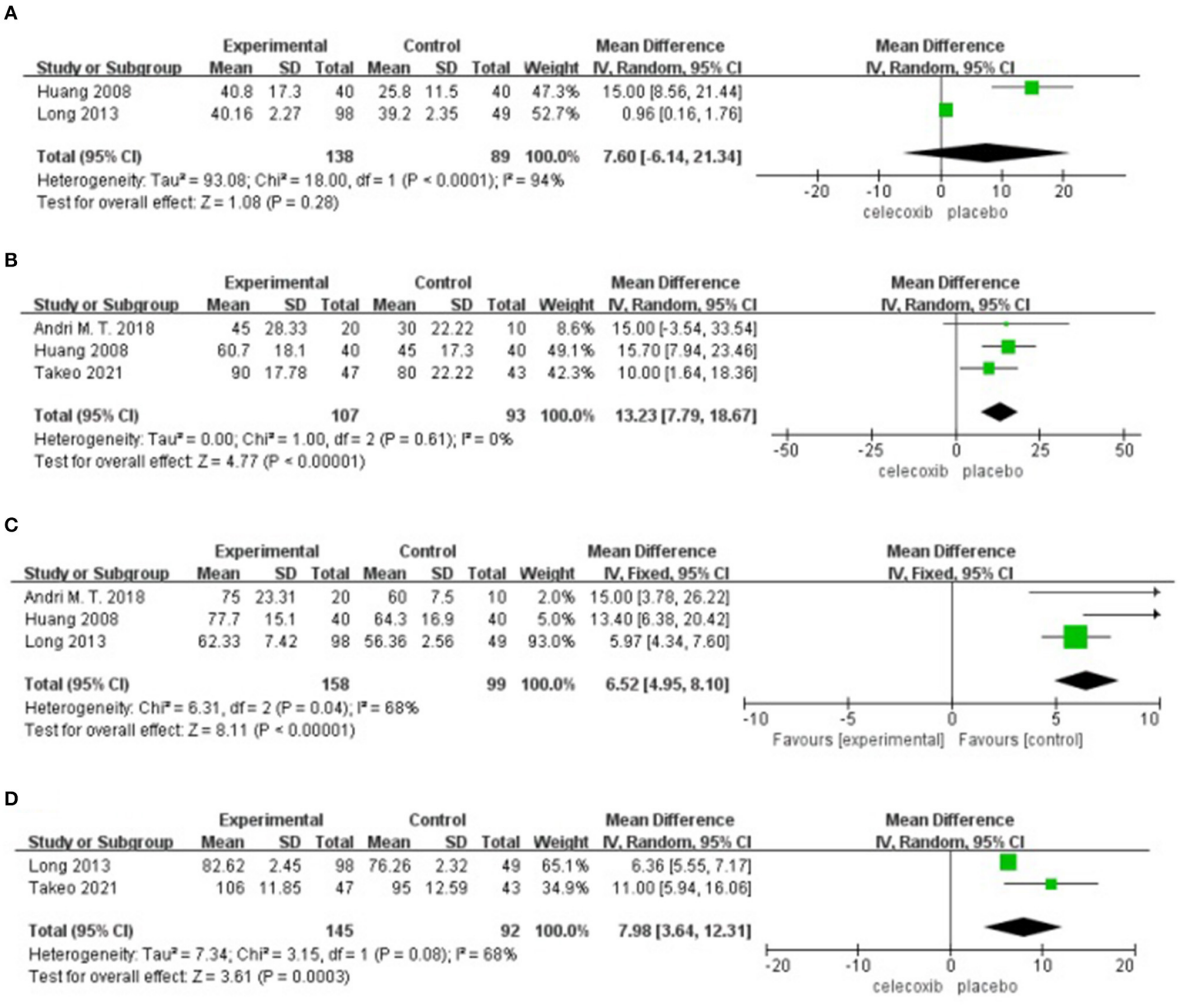
Forest plot for the meta-analysis of active ROM at postoperative **(A)** 24 h, **(B)** 48 h, **(C)** 72 h, and **(D)** 7 days.

Two studies (14, 16) reported active ROM at 24 h postoperatively. No significant difference was found in the active ROM at 24 h in the celecoxib group compared with the placebo group (MD = 7.60; 95% CI, −6.14 to 21.34; *I*^2^ = 94%; *P* = 0.28) (Figure 5A).

### Active ROM at Postoperative 48 h

Three studies (8, 13, 16) reported active ROM at 48 h postoperatively. Pooled results showed a significant increase in the active ROM at 48 h in the celecoxib group compared with the placebo group (MD = 13.23; 95% CI, 7.79 to 18.67; *I*^2^ = 0%; *P* < 0.00001) (Figure 5B).

### Active ROM at Postoperative 72 h

Three studies (13, 14, 16) reported active ROM at 72 h postoperatively. Pooled results showed a significant increase in the active ROM at 72 h in the celecoxib group compared with the placebo group (MD = 6.52; 95% CI, 4.95 to 8.10; *I*^2^ = 68%; *P* < 0.00001) (Figure 5C).

### Active ROM at Postoperative 7 Days

Two studies (8, 14) reported active ROM on postoperative day 7. Pooled results showed a significant increase in the active ROM at 7 days in the celecoxib group compared with the placebo group (MD = 7.98; 95% CI, 3.64 to 12.31; *I*^2^ = 68%; *P* = 0.0003) (Figure 5D).

### The Incidence of PONV

**Figure 6:**
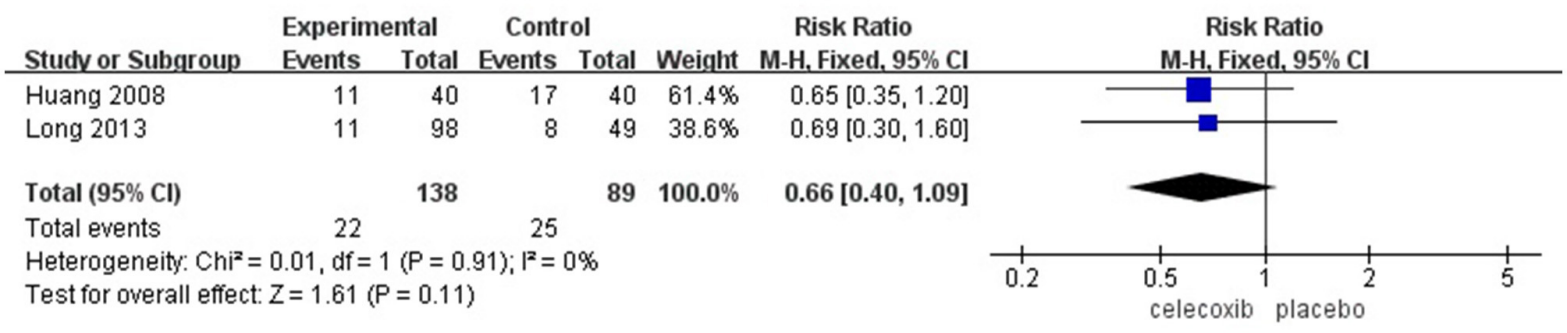
Meta-analysis of the incidence of PONV.

Two studies (14, 16) reported the incidence of PONV. No significant difference was found in the incidence of PONV in the celecoxib group compared with the placebo group (RR = 0.66; 95% CI, 0.40 to 1.09; *I*^2^ = 0%; *P* = 0.11) (Figure 6).

### Total Blood Loss After TKA

One study (14) reported total blood loss after TKA. No significant difference was found in total blood loss in the celecoxib group compared with the placebo group (MD = −34.0; 95% CI, −125.10 to 57.10; *P* = 0.46).

### Publication Bias

Funnel plots for publication bias could not be reliably tested because the number of included studies was small.

## Discussion

This meta-analysis showed that the perioperative use of celecoxib at a dose of 200–400 mg can reduce the intensity of postoperative pain at rest at 24, 48, and 72 h after TKA. Morphine consumption over 72 h postoperatively was also significantly reduced in the celecoxib group. Moreover, celecoxib significantly improved the postoperative active ROM at 48, 72 h, and 7 days after TKA. However, celecoxib had no significant effect on PONV and blood loss.

Enhanced recovery after surgery (ERAS), first proposed by Kehlet (17), is widely used in most surgical fields. This study aimed to optimize traditional perioperative care to reduce postoperative morbidity, shorten the length of hospital stay, and accelerate postoperative recovery (17). Because of the weight-bearing characteristics of the knee joint and the high demand for functional exercise after surgery, TKA has been regarded as the most painful type of orthopedic surgery (18). Furthermore, most of the patients who underwent TKA belong to middle-aged and elderly populations; therefore, the excessive use of morphine may have easily induced addiction and caused obvious side effects, especially in elderly patients with cardiopulmonary diseases (19). Therefore, pain management for patients undergoing TKA remains difficult and has attracted wide attention (13).

Celecoxib has been proven to be beneficial for pain control in various orthopedic surgical interventions (20). Patients receiving 300 mg of celecoxib twice daily for 14 days after TKA have significantly decreased postoperative VAS scores on ambulation, reduced morphine consumption, and improved postoperative ROM without experiencing an increased estimated postoperative blood loss (14). In another study, a decrease in opioid consumption, lower occurrence of adverse events, lower VAS pain scores, and higher Knee Society Scores and EQ-5D scores were associated with celecoxib use for TKA (15).

The VAS score is the most common assessment tool for pain intensity. As the primary outcome in our meta-analysis, data on VAS scores showed that celecoxib was associated with effective pain relief, which is consistent with the findings of previous studies (20). Meanwhile, a meta-analysis involving 548 patients who underwent arthroscopy revealed that the administration of celecoxib (200 or 400 mg) before surgery reduced pain intensity without increasing the incidence of PONV (21). Our results indicate that the perioperative use of celecoxib can significantly reduce VAS scores at rest at 24, 48, and 72 h after TKA. Therefore, we recommend the perioperative use of celecoxib as routine analgesia for TKA in the absence of any contraindication.

Early mobilization after TKA may contribute to better outcomes, such as lower morbidity and shorter hospital length of stay (22). Thus, active ROM assessment is an important indicator for evaluating postoperative recovery after TKA. However, many factors may affect postoperative mobilization, such as age, comorbidities, muscle strength, and psychological factors (15). Our meta-analysis revealed that the administration of celecoxib significantly increased active ROM at postoperative 48 h and 7 days postoperatively but not at 24 h, which may be due to early postoperative edema; however, further studies are needed to verify this.

Nausea and vomiting are among the most common postoperative complications, which may lead to dehydration, electrolyte imbalance, and prolonged hospital stay (23). The recommended pharmacologic antiemetics for PONV include 5-hydroxytryptamine (5-HT_3_) receptor antagonists, such as antagonists, dolasetron and corticosteroids, antihistamines, and anticholinergics (24). The perioperative administration of celecoxib decreased the incidence of PONV due to pain relief and reduced the consumption of morphine after TKA; however, our results showed that celecoxib had no significant effects on the incidence of PONV. Further studies with larger sample sizes are needed to confirm this.

Nevertheless, the results of several out outcomes from included studies were not inconsistent with our pooled results. For example, one study (13) showed that there was no difference in VAS scores at rest at postoperative 24 h between the placebo and celecoxib group, which may be due to the timing of administration. In another study (14), no difference in active ROM at postoperative 48 h between the placebo and celecoxib group which may be influenced by the small sample. Therefore, our pooled results regarding some outcomes may be underestimated by several included studies. Large sample and well-designed RCTs are needed.

This meta-analysis has several limitations. First, only five studies were included, and the sample size was relatively small. Particularly, one study only had a sample size of 30. Second, there was significant heterogeneity among the included studies, which might have resulted from different surgical procedures, doses, and timing of celecoxib use. Therefore, the pooled results from these included studies should be treated with caution. Large samples and multicenter RCTs are still needed to determine the optimal strategy of celecoxib use for postoperative pain after TKA.

## Conclusions

This meta-analysis demonstrated that perioperative use of celecoxib decreased pain scores at 24, 48, and 72 h postoperatively and increased active ROM at 48, 72 h, and 7 days postoperatively. We therefore recommend the routine perioperative use of celecoxib as a part of multimodal analgesia after TKA to enhance postoperative recovery.

## Data Availability Statement

The original contributions presented in the study are included in the article/supplementary material, further inquiries can be directed to the corresponding author/s.

## Author Contributions

XG and DZ designed and conceived the study, performed the statistical analysis, and drafted the manuscript. SZ and XZ performed the systematic review, study selection, statistical analysis, and preparation of the article for publication. XL and LJ contributed to data extraction and quality assessment. All authors participated in writing the manuscript, preparing the figures, have read, and approved the final manuscript.

## Conflict of Interest

The authors declare that the research was conducted in the absence of any commercial or financial relationships that could be construed as a potential conflict of interest.

## Publisher's Note

All claims expressed in this article are solely those of the authors and do not necessarily represent those of their affiliated organizations, or those of the publisher, the editors and the reviewers. Any product that may be evaluated in this article, or claim that may be made by its manufacturer, is not guaranteed or endorsed by the publisher.
